# Elevated Serum Ferritin Level Is Associated with the Incident Type 2 Diabetes in Healthy Korean Men: A 4 Year Longitudinal Study

**DOI:** 10.1371/journal.pone.0075250

**Published:** 2013-09-30

**Authors:** Chang Hee Jung, Min Jung Lee, Jenie Yoonoo Hwang, Jung Eun Jang, Jaechan Leem, Joong-Yeol Park, JungBok Lee, Hong-Kyu Kim, Woo Je Lee

**Affiliations:** 1 Department of Internal Medicine, Asan Medical Center, University of Ulsan College of Medicine, Seoul, Republic of Korea; 2 Department of International Healthcare Center, Asan Medical Center, University of Ulsan College of Medicine, Seoul, Republic of Korea; 3 Department of Clinical Epidemiology and Biostatistics, Asan Medical Center, University of Ulsan College of Medicine, Seoul, Republic of Korea; 4 Department of Health Screening and Promotion Center, Asan Medical Center, University of Ulsan College of Medicine, Seoul, Republic of Korea; FuWai hospital, Chinese Academy of Medical Sciences, China

## Abstract

**Background:**

Elevated ferritin concentration has been implicated in the etiology of type 2 diabetes. Accumulating evidence, mostly from studies conducted on western populations, has demonstrated a strong association between the elevated ferritin concentrations and incident type 2 diabetes. In Asian populations, however, the longitudinal studies investigating the association of elevated serum ferritin levels and type 2 diabetes are lacking. In present study, we aimed to determine whether elevated serum ferritin levels are related to the incident type 2 diabetes in healthy Korean men.

**Methodology/Principal Findings:**

This 4 year longitudinal observational study was conducted at the Asan Medical Center, Seoul, Republic of Korea. The study population consisted of 2,029 men without type 2 diabetes who underwent routine health examination in 2007 (baseline) and 2011 (follow-up). Baseline serum ferritin concentrations were measured by chemiluminescent two-site sandwich immunoassay. In multiple-adjusted model, the relative risk (RR) for incident type 2 diabetes was significantly higher in highest compared with the lowest ferritin quartile category, even after adjusting for confounding variables including homeostasis model assessment of insulin resistance (RR = 2.17, 95% confidence interval 1.27–3.72, *P* for trend = 0.013).

**Conclusions/Significance:**

These results demonstrated that elevated level of serum ferritin at baseline was associated with incident type 2 diabetes in an Asian population.

## Introduction

Iron is a transitional metal and micronutrient which is essential for several physiological functions in the body. Iron is also a potent pro-oxidant known to catalyze the formation of reactive oxygen species [1]. Excessive iron stores have been suggested to be associated with higher risk of metabolic disorders including hypertension [2], metabolic syndrome [3], and cardiovascular disease [4]. In addition, high iron stores have been proposed to contribute to the development of type 2 diabetes by causing pancreatic beta cells damage and insulin resistance through heightening the level of oxidative stress [5].

Ferritin, a key protein regulating iron homeostasis, is a widely used parameter to evaluate iron homeostasis in the body [6]. Based on the observation that type 2 diabetes is commonly complicated in patients with hereditary hemochromatosis, which is characterized by extremely high levels of circulating ferritin [1], several clinical studies have investigated the association of increased serum ferritin levels with an increased risk of future type 2 diabetes [6]–[12]. However, the results were inconsistent between different populations [6]–[12]. In the background of these inconsistence between studies, three meta-analysis on the positive association between the elevated serum ferritin levels and the risk of type 2 diabetes have been recently released [13]–[15]. However, they mostly included studies conducted in Western populations. Among them, only one meta-analysis included studies conducted in Asian populations [13]. Although all of those studies in Asian populations have reported the consistent positive association between elevated serum ferritin levels and the risk of type 2 diabetes, they were designed in a cross-sectional nature [16]–[20], through which the temporal relationship between exposure and outcome cannot be assessed [21].

Although we could not know the reason why the results were inconsistent between different populations, the several possibilities were the followings. Serum ferritin concentrations are variable among different ethnicities [22]. In a previous observational study called as Hemochromatosis and Iron Overload Screening Study (HEIRS) Study, Asians had higher serum ferritin levels compared to whites, indicating the innate biological differences across ethnic groups [22]. In case of Asian men, they had a 69 ng/ml higher adjusted mean serum ferritin levels compared with their white counterparts [22]. In addition, different body composition according to the ethnicity has been suggested to have a diverse effect on the association between serum ferritin and the insulin resistance [3], [23], [24]. Thus, it still remains unclear whether elevated serum ferritin concentration contributes to incident type 2 diabetes in Asian, especially in Korean.

In light of these findings, we designed this study to investigate the longitudinal effects of baseline serum ferritin concentrations on incident type 2 diabetes during a 4 year follow-up period in middle aged Korean Men.

## Materials and Methods

### Ethics Statement

All enrolled subjects provided written informed consent, and this study was approved by the Institutional Review Board of the Asan Medical Center (AMC, Seoul, Republic of Korea).

### Study Subjects

**Figure 1:**
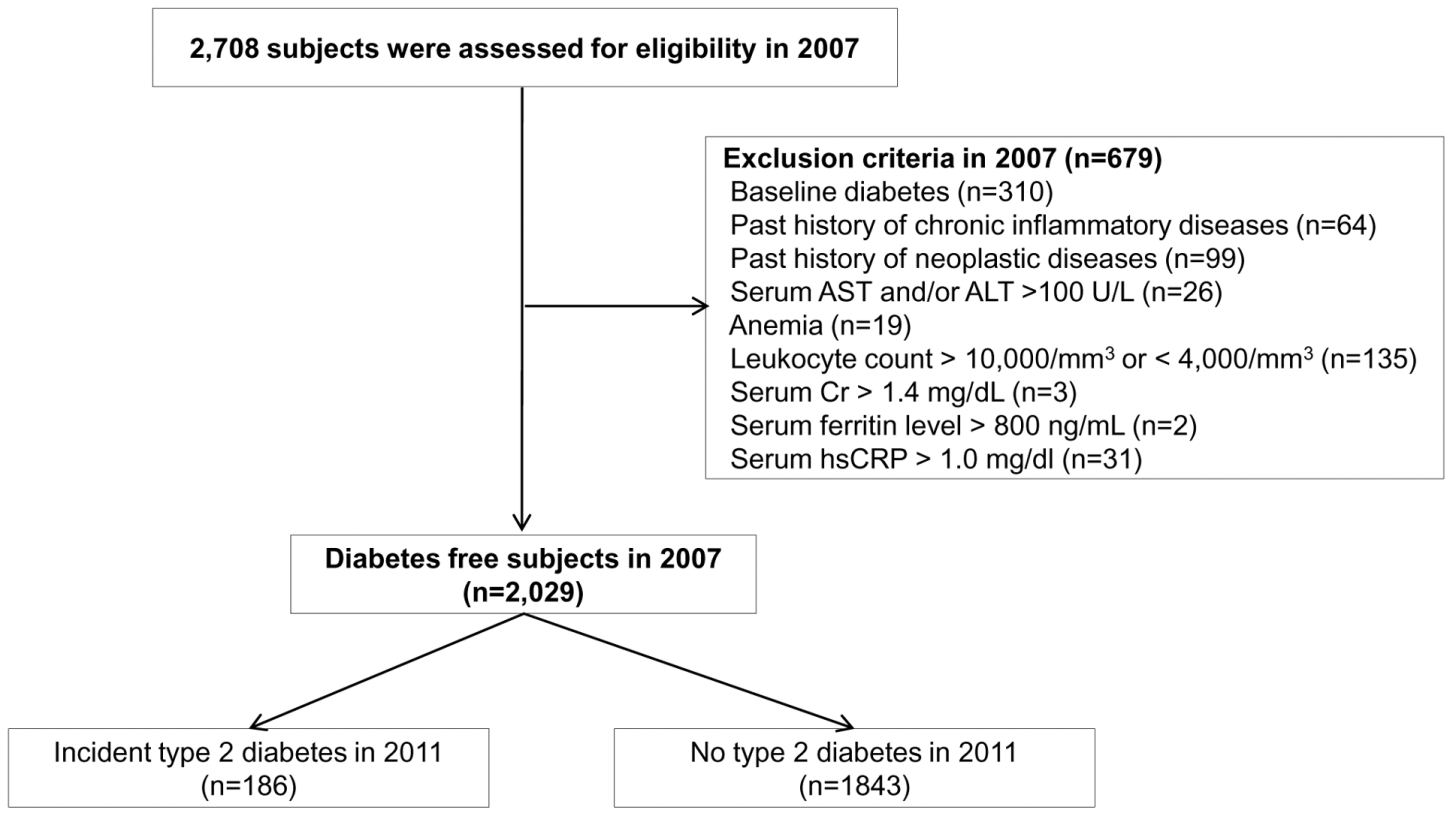
Selection of study participants.

The study cohort was a consecutive population of Korean men who had undergone comprehensive routine health examinations, including measurements of serum ferritin at the Health Screening and Promotion Center of the AMC in 2007 and returned for follow up examinations in 2011. All subjects visited the AMC health promotion center spontaneously for a routine health examination. This health promotion center has provided extensive screening tests, including blood cell counts and blood chemistries; urine analysis; chest radiographs; abdominal, gynecological, and thyroid ultrasound; mammography; duodenofiberscopy; and colonoscopy/barium enema. Visitors are usually healthy and receive the tests for early detection of malignancy, diabetes, and other age-related diseases. Initially, a total of 2,708 male Koreans were identified. Among these subjects, 310 were excluded due to baseline diabetes. Based on 2007 medical records, subjects were excluded for the following reasons: history of chronic inflammatory or infectious disease (n = 64), neoplastic disease (n = 99), abnormal liver enzyme levels (aspartate aminotransferase, AST and/or alanine aminotransferase, ALT≥2.5×upper limit of normal value; n = 19), anemia as defined as hemoglobin concentration less than 13.0 g/dL (n = 25), leukocytosis or leukopenia (blood leukocyte count>10.0×10^3^/mm^3^ or <4.0×10^3^/mm^3^; n = 135) and/or increased serum creatinine (>1.4 mg/dl; n = 3). Subjects with exceptionally high serum ferritin levels (>800 ng/ml; n = 2) were excluded to rule out those who could potentially have hemochromatosis [25]. Finally, subjects with high-sensitivity C-reactive protein (hsCRP) greater than 1.0 mg/dl (n = 19) were excluded to exclude occult infection or other systemic inflammatory process. Several subjects met more than two criteria. After exclusion of ineligible subjects, 2,029 male subjects with a mean age of 51.2 years (range, 23–82 years) were eligible for the study (Figure 1). As all subjects completed the follow-up visit in 2011, the follow-up durations were identical. Each subject completed a questionnaire listing medications, history of previous medical and/or surgical diseases, and drinking, smoking, and exercise habits. Drinking habit was defined as frequencies per week, i.e., ≤3 times/week, and ≥4 times/week; smoking habit as never, previous, or current; exercise habits as frequencies per week, i.e., ≤2 times/week and ≥3 times/week [26], [27].

### Clinical and Laboratory Measurements

Height (m) and weight (kg) were measured while subjects were wearing light clothing without shoes. Body mass index (BMI) was calculated as weight in kilograms divided by the square of height in meters. Waist circumference (WC, cm) was measured midway between the costal margin and the iliac crest at the end of a normal expiration. Blood pressure (BP) was measured using an automatic manometer with an appropriate cuff size on the right arm after a resting period of ≥5 min.

After overnight fasting, early morning blood was drawn from the antecubital vein into vacuum tubes and subsequently analyzed by a central, certified laboratory at the AMC. The department of Laboratory Medicine at AMC has been participating in the College of American Pathology (CAP) surveys since 1997 as well as undergoing CAP inspections since 1999. The laboratory has been also certified as a reference lab in clinical chemistry by participating in the Korean Association of quality assurance program. The concentrations of fasting plasma glucose (FPG), hemoglobin A1c (HbA1c), insulin, hsCRP, and concentrations of lipid components, and liver enzymes were determined.

Serum ferritin concentrations were determined by direct chemiluminescent two-site sandwich immunoassay using an ADVIA Centaur (Siemens Healthcare Diagnostics, Deerfield, IL, USA). Fasting total cholesterol, high density lipoprotein-cholesterol (HDL-C), low density lipoprotein-cholesterol (LDL-C), triglycerides (TG), uric acid, AST, and ALT levels were measured by an enzymatic colorimetric method using a Toshiba 200FR Neo autoanalyzer (Toshiba Medical System Co., Ltd., Tokyo, Japan). Gamma-glutamyltransferase (GGT) was measured using the L-γ-glutamyl-p-nitroanilide method (Toshiba). HsCRP and FPG concentrations were measured using an immunoturbidimetric method (Toshiba) and an enzymatic colorimetric method using a Toshiba 200FR autoanalyzer, respectively. Serum insulin concentrations were determined by an immunoradiometric assay (TFB Co., Ltd, Tokyo, Japan). Ion-exchange high-performance liquid chromatography (Bio-Rad Laboratories, Inc., Hercules, CA, USA) was used to measure HbA1c levels. The intra- and inter-assay coefficients of variations (CVs) of these analyses were consistently <3.5%. Homeostasis model assessment of insulin resistance (HOMA-IR) was calculated as the product of fasting serum insulin (µU/mL) and FPG (mg/dl) concentrations, divided by 405. All enzyme activities were measured at 37°C.

### Definition of Incident Type 2 Diabetes

Incident type 2 diabetes was determined by either FPG≥126 mg/dl or HbA1c level≥6.5% [27]–[29]. In addition, subjects who reported the use of antidiabetic medications on a self-report questionnaire at the final visit were considered to have incident type 2 diabetes during the 4 year period [27].

### Statistical Analyses

Continuous variables with normal distributions are expressed as the mean ± SD, whereas continuous variables with skewed distributions are expressed as median (and interquartile range). Categorical variables are expressed as proportions (%). Demographic and biochemical characteristics of the study population with respect to incident type 2 diabetes were compared using independent t-test or the Mann-Whitney U test for continuous variables and the chi-squared test for categorical variables. Characteristics of the study population according to the ferritin quartile categories were compared using one-way analysis of variance (ANOVA) or the Kruskal-Wallis test for continuous variables and the chi-squared test for categorical variables. After adjusting for confounding variables, multivariate logistic regression analysis was used to calculate the relative risks (RRs) of the ferritin quartile categories for incident type 2 diabetes. All statistical analyses were performed using SPSS version 17.0 for Windows (SPSS Inc., Chicago, IL). A *P* value of <0.05 was considered statistically significant.

## Results

Baseline clinical and biochemical characteristics of 2,029 subjects, including those who developed type 2 diabetes during the 4 year period, are presented in Table 1. At baseline, the mean (SD) age and serum ferritin levels of study participants were 51.2 years (8.4) and 160.0 ng/ml (91.5), respectively. During the 4 year period, 186 cases of type 2 diabetes (9.2%) were identified. The clinical and biochemical characteristics of the subjects with respect to incident type 2 diabetes are shown in Table 1. Subjects who subsequently developed type 2 diabetes were more likely to be older. They also had higher baseline BMI, WC, FPG, HbA1c, HOMA-IR, TG, liver enzymes (i.e., AST, ALT, and GGT), hsCRP, and ferritin levels, but a lower HDL-C than those who did not develop type 2 diabetes. There were no significant differences in the family history of diabetes or smoking, drinking and exercise habits between the two groups.

**Table 1:** Baseline clinical and biochemical characteristics of study subjects with respect to incident diabetes during a 4 year period.

	Incident type 2 diabetes			
	No	Yes		
n (%)	1843 (90.8)	186 (9.2)	*P* value	Overall
Age (yr)	50.9±8.3	54.4±8.3	<0.001	51.2±8.4
BMI (kg/m^2^)	24.6±2.8	25.8±3.6	<0.001	24.7±2.9
WC (cm)	84.5±11.8	88.2±10.2	<0.001	84.8±11.7
Systolic BP (mmHg)	121.5±13.1	121.6±16.5	0.961	121.6±13.4
Diastolic BP (mmHg)	75.5±8.4	75.7±9.0	0.714	75.5±8.5
Current smoker (%)	30.3	35.5	0.111	30.8
Drinking habits (%)^a^	76.6/23.4	73.1/26.9	0.279	76.3/23.7
Exercise habits (%)^b^	48.3/51.7	49.5/50.5	0.817	48.4/51.6
Family historyof diabetes (%)	21.3	18.8	0.452	21.2
FPG (mg/dl)	96.2±9.4	109.7±12.4	<0.001	97.3±10.3
HbA1c (%)	5.4 (5.1–5.6)	5.8 (6.0–6.2)	<0.001	5.2 (5.4–5.7)
HOMA-IR	1.7±0.9	2.2±1.2	<0.001	1.8±1.0
Total cholesterol(mg/dl)	193.6±31.6	192.7±37.6	0.758	193.5±32.2
TG (mg/dl)	123 (89–167)	141 (99–198)	<0.001	125 (90–170)
LDL-C (mg/dl)	126.8±28.8	125.3±33.3	0.553	126.6±29.2
HDL-C (mg/dl)	53.3±12.4	50.6±12.2	0.004	53.1±12.4
Uric acid (mg/dl)	5.9±1.2	6.0±1.2	0.704	5.9±1.2
AST (U/L)	21 (24–30)	26 (21–34)	0.010	25 (21–30)
ALT (U/L)	23 (17–31)	25 (19–36)	0.003	23 (18–32)
GGT (U/L)	25 (17–42)	32 (22–51)	<0.001	26 (18–42)
hsCRP (mg/dl)	0.07(0.05–0.12)	0.08(0.05–0.13)	0.026	0.07(0.05–0.13)
Ferritin (mg/dl)	157.4±89.1	185.8±109.4	0.001	160.0±91.5

BMI, body mass index; WC, waist circumference; BP, blood pressure; FPG, fasting plasma glucose; HbA1c, hemoglobin A1c; HOMA-IR, homeostasis model of insulin resistance; TG, triglycerides; LDL-C, low density lipoprotein-cholesterol; HDL-C, high density lipoprotein-cholesterol; AST, aspartate aminotransferase; AST, alanine aminotransferase; GGT, gamma-glutamyltransferase; hsCRP, high sensitive C-reactive protein.

a Represented in the following order; ≤3 times/week, ≥4 times/week.

b Represented in the following order; ≤2 times/week, ≥3 times/week.

Baseline clinical and biochemical characteristics for 2,029 subjects based on ferritin quartile categories are shown in Table 2. As the ferritin quartile increased, subjects were more likely to be younger and frequent drinkers. Positive relationships between ferritin quartiles and BMI, WC, systolic BP, diastolic BP, FPG, HOMA-IR, TG, uric acid, liver enzymes and hsCRP levels were observed, while a negative relationship existed with the HDL-C levels. Although there was no significant difference in the family history of diabetes, the incidence of type 2 diabetes increased across ferritin quartile categories (*P* = 0.003).

**Table 2:** Baseline clinical and biochemical characteristic of study subjects based on serum ferritin quartile categories.

	Q1 (≤97.2 IU/L)	Q2 (97.3–142.6 IU/L)	Q3 (142.7–200.5 IU/L)	Q4 (≥200.6 IU/L)	*P* value
n (%)	510 (25.1)	505(24.9)	508 (25.0)	506 (24.9)	<0.001
Age (yr)	52.6±8.4	51.2±8.1	50.7±8.2	50.3±8.6	<0.001
BMI (kg/m^2^)	24.3±2.5	24.6±2.6	24.7±3.5	25.3±2.7	<0.001
WC (cm)	83.1±12.7	84.3±12.4	85.0±10.9	86.8±10.4	0.003
Systolic BP (mmHg)	120.7±13.2	121.5±13.1	120.6±12.7	123.4±14.5	<0.001
Diastolic BP (mmHg)	74.8±8.5	75.5±8.4	75.0±8.7	76.9±8.2	0.867
Current smoker (%)	29.0	32.5	30.1	31.4	<0.001
Drinking habits (%)^a^	82.5/17.5	78.0/22.0	75.0/25.0	69.6/30.4	0.072
Exercise habits (%)^b^	52.4/47.6	48.7/51.3	48.4/51.6	44.1/55.9	0.914
Family history ofdiabetes (%)	21.6	20.2	21.9	20.8	0.015
FPG (mg/dl)	96.3±9.8	97.0±10.5	97.7±10.5	98.2±10.2	0.786
HbA1c (%)	5.4 (5.2–5.7)	5.4 (5.2–5.6)	5.4 (5.1–5.7)	5.4 (5.2–5.7)	<0.001
HOMA-IR	1.6±0.8	1.7±0.8	1.8±1.1	2.0±1.0	0.342
Total cholesterol (mg/dl)	192.8±35.2	191.7±30.0	194.7±30.7	194.8±32.6	<0.001
TG (mg/dl)	111 (84–156)	124 (90–166)	123 (89–169)	139 (100–196)	0.972
LDL-C (mg/dl)	126.4±31.1	126.3±28.9	127.1±27.3	126.8±29.4	0.023
HDL-C (mg/dl)	54.0±12.7	52.4±12.0	53.8±12.6	52.1±12.2	0.014
Uric acid (mg/dl)	5.8±1.2	5.9±1.2	6.0±1.2	6.1±1.2	<0.001
AST (U/L)	23 (20–28)	23 (20–29)	25 (20–30)	27 (22–34)	<0.001
ALT (U/L)	21 (16–27)	22 (17–29)	23 (18–31)	28 (21–40)	<0.001
GGT (U/L)	21 (15–32)	23 (17–41)	27 (18–41)	35 (23–57)	0.003
hsCRP (mg/dl)	0.06(0.04–0.12)	0.07 (0.04–0.12)	0.07 (0.05–0.12)	0.08 (0.05–0.14)	–
Ferritin (mg/dl)	68.4±21.1	119.3±12.9	169.0±16.1	283.7±84.9	0.003
Incident diabetes (n, %)	39 (7.6)	37 (7.3)	43 (8.5)	67 (13.2)	

BMI, body mass index; WC, waist circumference; BP, blood pressure; FPG, fasting plasma glucose; HbA1c, hemoglobin A1c; HOMA-IR, homeostasis model of insulin resistance; TG, triglycerides; LDL-C, low density lipoprotein-cholesterol; HDL-C, high density lipoprotein-cholesterol; AST, aspartate aminotransferase; AST, alanine aminotransferase; GGT, gamma-glutamyltransferase; hsCRP, high sensitive C-reactive protein.

a Represented in the following order; ≤3 times/week, ≥4 times/week.

b Represented in the following order; ≤2 times/week, ≥3 times/week.

The risk of developing type 2 diabetes based on baseline quartile groups of serum ferritin levels is shown in Table 3. In the unadjusted model, the RRs and 95% confidence interval (CI) for incident type 2 diabetes comparing the second quartile to the fourth quartile versus the first quartile of serum ferritin were 0.96 (0.60–1.52), 1.12 (0.71–1.76), and 1.84 (1.22–2.79), respectively (*P* for trend = 0.004). The associations also remained significant even after adjusting for confounding variables in models 1, 2, 3, 4, and 5 as shown in Table 3. In multivariate logistic regression models adjusting for age, HbA1c, WC, systolic BP, diastolic BP, drinking, smoking and exercise habits, family history of diabetes, hsCRP, GGT, AST, ALT, TG, HDL-C, LDL-C and HOMA-IR, the adjusted RRs and 95% CI for incident type 2 diabetes across baseline quartile groups of serum ferritin levels were 1.10 (0.63–1.93), 1.16 (0.67–1.99), and 2.17 (1.27–3.72), respectively (*P* for trend = 0.013).

**Table 3:** Relative risks (RRs) and 95% confidence intervals (CI) for incident type 2 diabetes based on serum ferritin quartile categories during a 4 year period.

	RR (95% CI)				
Subgroup	Q1 (≤97.2 IU/L)	Q2 (97.3–142.6 IU/L)	Q3 (142.7–200.5 IU/L)	Q4 (≥200.6 IU/L)	*P* for trend
Type 2 diabetes					
[no/total no. (%)]	39/510 (7.6)	37/505 (7.3)	43/508 (8.5)	67/506 (13.2)	
Unadjusted	1	0.96 (0.60–1.52)	1.12 (0.71–1.76)	1.84 (1.22–2.79)	0.004
Model 1	1	1.09 (0.62–1.89)	1.18 (0.69–2.02)	2.30 (1.37–3.85)	0.003
Model 2	1	1.11 (0.64–1.94)	1.18 (0.69–2.02)	2.36 (1.41–3.96)	0.002
Model 3	1	1.11 (0.63–1.93)	1.19 (0.69–2.04)	2.18 (1.28–3.71)	0.012
Model 4	1	1.07 (0.61–1.87)	1.16 (0.67–1.99)	2.25 (1.34–3.79)	0.004
Model 5	1	1.10 (0.63–1.93)	1.16 (0.67–1.99)	2.17 (1.27–3.72)	0.013

Model 1: adjusted for age, HbA1c, WC, systolic BP, diastolic BP, drinking, smoking, exercise habits, and family history of diabetes.

Model 2: adjusted for variables in Model 1 plus hsCRP.

Model 3: adjusted for variables in Model 1 plus GGT, AST and ALT.

Model 4: adjusted for variables in Model 1 plus TG, HDL-C, LDL-C, and HOMA-IR.

Model 5: adjusted for overall confounders noted above.

## Discussion

In this longitudinal observational study, we observed a positive and significant association between elevated basal serum ferritin levels and incident type 2 diabetes during a 4 year period in initially diabetes-free Korean men. This positive association remained significant after adjusting for conventional risk factors, inflammatory marker and liver function. This finding suggests that ferritin plays a significant role in incident diabetes in Asian populations similar to western populations [6]–[8], [11], [12].

The role of elevated serum ferritin concentrations in incident type 2 diabetes in western populations has been investigated in several studies [6]–[12], however, prospectively designed study that examines this association in an Asian population was only one [30]. Health disparities in different ethnic populations have become a topic of intense research for the past few decades, yet they are still poorly understood [31]. In this background, we carried out this longitudinal study to determine whether an association similar to that observed in western populations exists. Our results are in line with the previous cross-sectional studies and extend earlier observations by examining the longitudinal association between baseline serum ferritin levels and the incident type 2 diabetes in an Asian population [16]–[20].

Previously, Zumin Shi et al., conducted a prospective study which showed that higher body iron stores measured by serum ferritin were associated with an increased risk of hyperglycemia in a Chinese population [30]. However, they included subjects with anemia, which might affect the serum ferritin levels [30]. Furthermore, the association became marginally significant when other confounding variables such as BMI were included [30]. Compared to that study, our study was conducted on more large number of Asian population and showed the significant association between elevated serum ferritin levels and incident type 2 diabetes even after adjusting for various known risk factors (Table 3). Furthermore, we excluded subjects with clinical conditions in which ferritin levels might be affected as much as we could.

Although the exact mechanism underlying the association between the elevated serum ferritin concentrations and incident type 2 diabetes is not known, several possible mechanisms have been suggested. One possible explanation is that iron deposition in the liver can result in hepatic insulin resistance and increase hepatic glucose production [8], [32], [33]. Furthermore, other studies have suggested a link between serum ferritin and nonalcoholic fatty liver disease, currently regarded as one of the independent risk factors for incident type 2 diabetes [34], [35]. Although we did not obtain any information on fatty liver disease in our population, we did observe a positive relationship between the ferritin quartile groups and liver enzymes (i.e., ALT, AST, and GGT; Table 2). This finding is in line with previously published results [8], [17], and supports the above hypothesis. However, the association between the elevated ferritin levels at baseline and incident type 2 diabetes remained significant even after adjusting for liver enzymes (Table 3). These results suggest that other mechanisms might be involved in the association between the elevated ferritin levels and incident type 2 diabetes.

Another explanation for the relationship between elevated ferritin levels and incident type 2 diabetes involves an elevation in oxidative stress through the increased formation of hydroxyl radicals catalyzed by iron, which may lead to systemic insulin resistance and hyperglycemia [36]. In addition, iron excess probably contributes initially to insulin resistance and subsequently to decreased insulin secretion [37]. In our study, we observed a significant positive correlation between the level of ferritin, HOMA-IR and HOMA-β (correlation coefficient = 0.146 for HOMA-IR and 0.063 for HOMA-β, respectively; *P*<0.001 for HOMA-IR and *P* = 0.005 for HOMA- β, Figure S1). As the observational period of our study was relatively short (i.e., 4 years), the duration of diabetes is not accordingly long in our population. Considering the positive correlations described above, an increase in ferritin-related insulin resistance, rather than a decrease in insulin secretion, is likely to prevail during this early stage of type 2 diabetes.

Lastly, elevated ferritin concentrations might reflect systemic inflammation besides elevated body iron stores [38]. It is well known that inflammation is an important mediator of type 2 diabetes [39]. In line with this, we observed an increase in hsCRP levels coinciding with an increase in ferritin quartile (Table 2). However, the association between ferritin quartiles and increased risk for type 2 diabetes remained significant even after adjusting for confounding variables including hsCRP in our study (Table 3) and other previous studies [8], [9], [11], [12]. This might implicate that ferritin increases the risk of type 2 diabetes through another various mechanisms besides systemic inflammation. Chronic inflammation with insulin resistance is also known to be involved in the development of metabolic syndrome, a well known risk factor for type 2 diabetes and cardiovascular diseases [39]. Recently, Park et al. reported that elevated serum ferritin levels were independently associated with future development of metabolic syndrome during the a 5 year follow-up period in Koreans [25]. In our study, RRs for incident type 2 diabetes were still significant even after adjusting for components of metabolic syndrome (i.e., WC, systolic BP, diastolic BP, TG, and HDL-C; Table 3). These results further support the role of ferritin in the pathogenesis of type 2 diabetes independent of systemic inflammation.

In our study, RRs for incident type 2 diabetes was significant in the fourth ferritin quartile (i.e., ≥200.6 ng/ml) in every model (Table 3). This level was quite similar to the levels of previous cross-sectional studies conducted on Asian male populations [17], [20]. However, it was lower than the level of previous prospective study conducted on Western male population (i.e., ≥300 ng/ml) [8]. The reason for this discrepancy between different ethnic populations is unclear now. However, it seems that a threshold exists above which ferritin concentrations are associated with incident type 2 diabetes.

This study had several important limitations. Firstly, we could not ascertain that subjects were representative of the general Korean population since participants were voluntarily recruited during routine health examinations; thus, there remains a possibility of selection bias. Secondly, the lack of a 2 hour oral glucose tolerance test was another limitation of this study because it may have resulted in inclusion of subjects with undiagnosed diabetes at baseline. Thirdly, we examined only men; therefore, our results cannot be extrapolated to women whose serum ferritin levels are lower than those of men [40]. Fourthly, the self-reported questionnaire we used did not have a validation study. However, this questionnaire seemed to be somewhat reliable when we found that serum ferritin levels showed positive relationship between drinking and exercise habits (Table 2), which were in concordance with the results of previous studies [41], [42]. Lastly, we measured serum ferritin as the only marker of iron storage and did not quantify other markers of iron overload such as transferrin saturation. As mentioned earlier, ferritin is not an entirely specific marker for iron storage. It is also known as an acute-phase reactant and thus may be artificially elevated in response to systemic inflammation [38]. However, we tried to exclude subjects with comorbities and conditions which were associated with increased or decreased levels of ferritin as much as possible we could.

Despite these limitations, our study was robust in that we analyzed data from a relatively large number of Asian subjects and evaluated the risk of incident type 2 diabetes in individuals with elevated ferritin concentrations.

In conclusion, these results demonstrated that elevated level of serum ferritin at baseline was associated with incident type 2 diabetes in an Asian population.

## Supporting Information

Figure S1 **Correlation between serum ferritin and HOMA-IR (A), and HOMA-β (B).** The correlation analysis were performed using Pearson’s correlation analysis.
(DOCX)
