# Supplementary figures and images for: Elevated Serum Ferritin Level Is Associated with the Incident Type 2 Diabetes in Healthy Korean Men: A 4 Year Longitudinal Study
Source: PLoS One. 2013 Sep 30;8(9):e75250. doi: 10.1371/journal.pone.0075250 (PMC3787082; doi:10.1371/journal.pone.0075250)

**
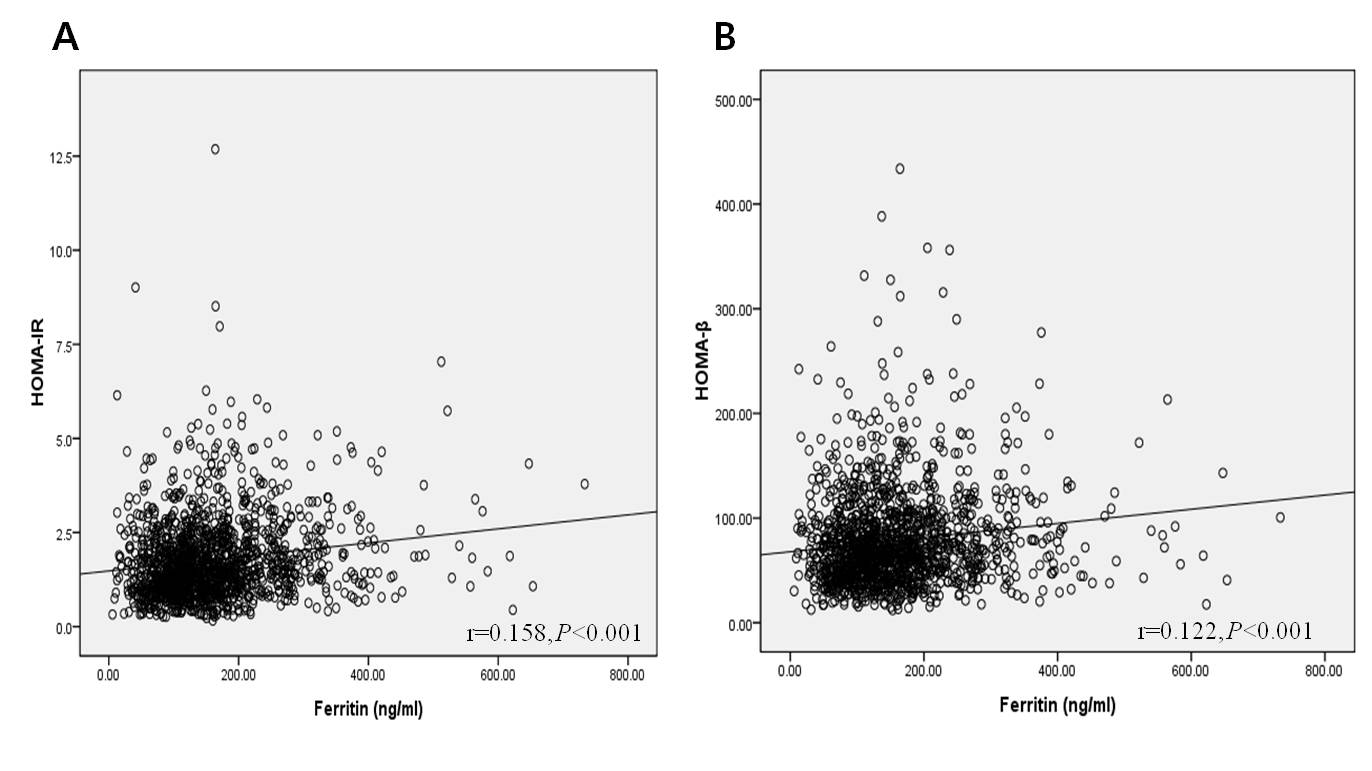
**

Supplement: Figure S1 — Correlation between serum ferritin and HOMA-IR (A), and HOMA-β (B). The correlation analysis were performed using Pearson’s correlation analysis. (DOCX) [file pone.0075250.s001.docx]
